# Evaluating Undergraduate Dental Curricula on Oral Health Care for Autistic Persons in Australia and New Zealand—A Cross-Sectional Study

**DOI:** 10.3390/dj14040238

**Published:** 2026-04-15

**Authors:** Jayne Jones, Dileep Sharma, Kuang-Yin Chu, Elysa Roberts, Deborah Cockrell

**Affiliations:** 1 Discipline of Oral Health, School of Health Sciences, College of Health, Medicine and Wellbeing, University of Newcastle, Ourimbah, NSW 2258, Australia; jayne.jones@newcastle.edu.au (J.J.); kuangyin.chu@newcastle.edu.au (K.-Y.C.); deborah.cockrell@newcastle.edu.au (D.C.); 2 Department of Periodontology, Dr. D. Y. Patil Dental College and Hospital, Dr. D. Y. Patil Vidyapeeth, Pune 411 018, India; 3 Faculty of Dentistry, SEGi University, Petaling Jaya 47810, Malaysia; 4 Discipline of Occupational Therapy, College of Health, Medicine and Wellbeing, University of Newcastle, Callaghan, NSW 2308, Australia; elysa.roberts@newcastle.edu.au

**Keywords:** autism spectrum disorder, dental education, curriculum, students, surveys and questionnaires, simulation training, Australia, New Zealand

## Abstract

**Introduction**: Persons diagnosed with Autism Spectrum Disorder (ASD) require adaptations to dental care that many undergraduate programmes may not explicitly treat. This cross-sectional pilot study assessed the extent of ASD-related content in Australia and New Zealand (ANZ) dental and oral health curricula and explored Oral Health Therapy students’ knowledge and self-efficacy. **Methods**: Online surveys of academic staff across ANZ programmes and Bachelor of Oral Health Therapy students at the University of Newcastle were conducted. Quantitative data was summarised descriptively, and free text responses underwent thematic analysis. **Results**: Fifteen educator responses (8% of 178 invitees) suggest limited ASD-specific teaching and minimal use of simulation-based education. Among 38 student responses (from one institution), knowledge was generally foundational, but misconceptions persisted and no respondents reported high confidence in providing oral health care for Autistic patients. Interest in further training was high. **Conclusions**: Within the constraints of low response rates and a single institution student sample, these preliminary findings suggest opportunities to strengthen Autism-related teaching, particularly sensory adaptations, communication strategies, and experiential learning. Inferences should be considered exploratory and hypothesis generating. **Limitations**: Low educator responses and potential response bias due to limited external validity from a single student cohort.

## 1. Introduction

The American Psychiatric Association defines Autism Spectrum Disorder (ASD) as “characterized by persistent deficits in social communication and social interaction across multiple contexts, accompanied by restricted, repetitive patterns of behaviour, interests, or activities” [1,2]. Worldwide, a significant increase in the number of people diagnosed with Autistic Spectrum Disorder (ASD) has been noted over the last 20 years, needing measures to improve the health and wellbeing of these affected individuals and their families [3]. The World Health Organisation (WHO) in 2018 reported that the childhood incidence of Autism Spectrum Disorder (ASD) worldwide was 1 in 160 [4]. However, in 2021 the incidence was reported as approximately 1 in 127 persons demonstrating an increasing incidence worldwide [4].

Beyond Autism-specific barriers, people with disabilities experience structural and environmental obstacles to oral health care, underscoring the need for disability-informed undergraduate training [5]. Recent evidence links greater exposure and education to improved student preparedness and attitudes towards treating persons with disabilities, and systematic reviews indicate that Autistic individuals often exhibit worse oral health indicators than neurotypical peers, highlighting the clinical relevance of Autism-related competencies within dental curricula [6].

Autism begins in childhood, is usually diagnosed by the age of 3 and can persist into adolescence and adulthood [1,4]. ASD affects approximately four times as many males as females [7] and as the name suggests it is a spectrum of disorders. The aetiology of ASD is unclear and with the lack of biological tests for ASD [7], diagnosis is based on specific behaviours relating to social communication and restrictive and repetitive behaviours and interests [8]. In Australia an estimated 290,900 people were diagnosed with Autism in 2022, which represented approximately 1.1% of the Australian population and a 41.8% increase from 2018 wherein the incidence was at 0.8% [9]. Due to this increase, student clinicians and dental practitioners are highly likely to encounter patients with ASD during their practicing career. Therefore, effective training and education in this domain is essential to ensure optimum clinical skills are developed during their coursework to develop competent practitioners.

Dental and oral health students receive training to adapt to the treatment needs of their patients with special needs. However, persons with Autism have reported to experience significant barriers to oral health care that can be related to inadequate education around adapting to their sensory needs in the traditional dental setting [7,10]. Evidence suggests that dental graduates are more likely to provide optimal dental treatment for persons with Autism when they gain education, experience, confidence and competence to deliver oral health care for this disadvantaged population as students [11,12]. Consequently, dental students need specialised training to assess or provide treatment for patients with Autism [10,13]. University dental clinics are well placed to provide a supportive environment wherein equitable person-centred care is delivered for patients of all abilities and sensitivities; therefore, it is essential that dental students complete supervised training and adapt to the special needs of patients.

This study aimed to evaluate the extent of Autism-related curricula in undergraduate dental programmes in Australia and New Zealand and to explore Oral Health Therapy students’ knowledge, confidence, and attitudes toward managing persons with Autism. By combining quantitative survey data with thematic analysis of open-ended responses, this research provides insights into current educational gaps and informs strategies for curriculum enhancement.

## 2. Methods

This study was approved by the Human Research Ethics Committee at the University of Newcastle (# H-2024-0070, approved 8 May 2024). The methodology employed in this study included quantitative surveys with some open-ended questions included.

Two groups of participants were involved in this study:

1. Educators recruited through the College of Oral Health Academics (COHA) and Australasian Council of Dental School (ACODS)
2. Bachelor of Oral Health Therapy (BOHT) students from University of Newcastle (UON).

The COHA and ACODS participants consist of educators teaching in ten oral health programmes in Australia and two in New Zealand. The student participants were those enrolled in the BOHT program at UON in 2024.

No power calculation was deemed necessary, given the exploratory and descriptive nature of this pilot study, so no power calculation was performed. The educator survey used voluntary participation and branching logic, resulting in variable denominators across items. Since only respondents who indicated that their curriculum included Autism-related content had access to subsequent items, a high proportion of “no reply” responses were expected. Therefore, all analyses planned were descriptive, and findings will need to be interpreted cautiously in light of the response rates and possibility of item-level missing data.

This study follows the STROBE guideline for cross-sectional studies and a completed checklist is provided as Supplementary Materials [14]. Data collection was completed between 3 October and 25 November 2024 using REDCap^®^, a secure, web-based platform for research data capture [15,16].

### 2.1. Educator Surveys

The main objective of the survey involving educators (Table 1) was to assess curricula around adapting to the treatment needs of persons with Autism. Educators from Australia and New Zealand were invited to participate in an online questionnaire (using RedCap^®^) on the current teaching activities in special needs dentistry, specifically around the treatment needs of persons with Autism. The online survey was distributed via the secretariat to members of the College of Oral Health Academics (COHA) and the Australasian Council of Dental Schools (ACODS). Participants were invited via email and completion of the questionnaire was considered as implied consent.

The questionnaire was designed to capture current content and teaching on the oral health care needs of persons with Autism, including the timing and mode of delivery in undergraduate education and training; awareness around sensory adaptations to the dental environment; utilisation of simulation-based education; and level of interest in the development of further education and training of undergraduate dental students. The survey consisted of polar questions (yes/no responses) and optional free text responses. The educator questionnaire was based on a previous study by Higgins et al. which documented the curriculum design and delivery of simulation-based education in oral health programmes in Australia and New Zealand [17]. To ensure the validity and reliability of the questionnaire, a panel of experts including educators and clinicians reviewed the survey questions.

### 2.2. Student Surveys

First and third year Bachelor of Oral Health Therapy students (2024 cohort) at the University of Newcastle were invited to participate in an online questionnaire (using RedCap^®^) to assess their knowledge of Autism and perceptions around managing persons with Autism. The BOHT student cohort was provided with the information statement and completed the online questionnaire voluntarily. Completion and submission of the survey was taken as implied consent. The survey responses were de-identified and provided an option for withdrawal from participation any time prior to submission of the completed questionnaire.

**Figure 1:**
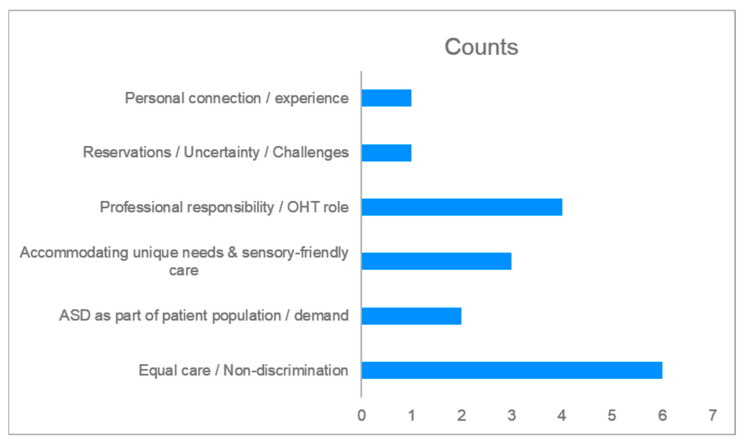
Identified themes and counts for Question 1: “*Do you plan to treat persons with ASD as an Oral Health Therapy graduate?*”.

**Figure 2:**
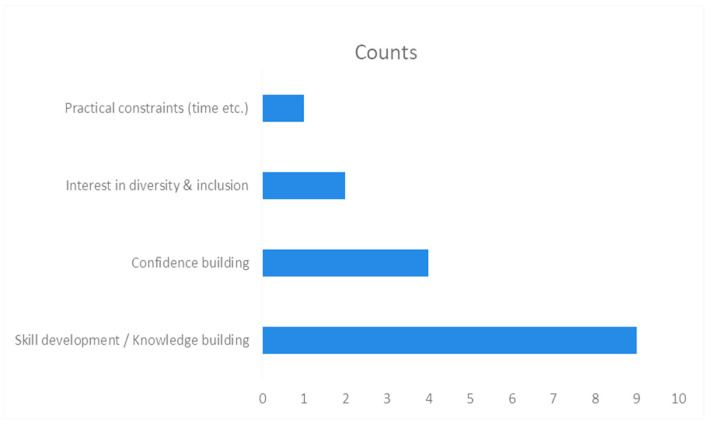
Identified themes and counts on Question 1: “*Do you plan to treat persons with ASD as an Oral Health Therapy graduate?*”.

The questionnaire utilised for the BOHT students’ cohort differed from the educators in that it captured their knowledge and experience of persons with Autism and their qualitative responses focused on their confidence in managing persons with Autism. The questionnaire was based on a previous study assessing dental practitioners’ knowledge and confidence when treating persons with Autism in the UK [18]. The first part of the questionnaire collected demographic characteristics (Table 2); the second part assessed knowledge on Autism (Table 3); the third part of the questionnaire assessed the self-efficacy scale of the dental students (Table 4); and the final part included two open-ended questions (Table 5 and Table 6, Figure 1 and Figure 2).

The quantitative data was analysed using IBM SPSS v29 with frequencies and percentages calculated for knowledge and confidence levels, which were grouped into three categories: low (score 1–3), moderate (4–6), and high (7). Responses to open-ended questions were analysed using reflexive thematic analysis following Braun and Clarke’s six-phase framework [19,20,21,22]. An inductive, primarily semantic approach was adopted to identify patterns across responses within a framework of interpretivism and constructivism. Themes were reviewed for coherence and defined with supporting quotes from students.

## 3. Results

### 3.1. Educator Surveys

The educator surveys conducted in this study assessed the current curricula in the teaching of special needs dentistry. Of the 178 COHA and ACODS members from universities across Australia and New Zealand invited to complete the educator surveys, 15 responses were received. Whilst this is a low response rate (8.4%), it represents responses from a range of universities across Australia and New Zealand.

The educator survey results (Table 1) indicate that 46.7% of the respondents reported that their curricula included teaching on managing persons with Autism; however, their delivery and approaches varied: face-to-face (33.3%); online synchronous (13.3%); online asynchronous (13.3%); and role play (6.7%). The majority of the respondents (93.4%) reported that their curriculum did not include simulation-based activities in preparing students for delivery of oral health care in persons with Autism. Additionally, 33.3% expressed interests in using a simulation-based education module, if available.

Educators were not able respond to all questions in the surveys since the survey logic only progressed to further details when they positively responded to the question if their teaching included Autism training. Only seven (46.7%) of the educators positively responded to the question *“Does your Dentistry or Oral Health curriculum include education for students in the treatment and management of persons with Autism?”*, three (20%) did not respond and five (33.3%) said no. Consequently, only 46.7% of the educators completed the section related to the details of curriculum content. Of the respondents, three (20.0%) reported familiarity with “*Sensory Adapted Dental Environment*” and two (13.3%) were not, whilst nine (66.7%) respondents did not choose to answer. Only one (6.7%) responded that they currently utilised simulation-based learning in the training for management of persons with Autism while two (13.3%) responded that they would consider simulation-based learning.

Overall, findings from this survey involving educators have suggested there is limited teaching in the curricula focused on adapting to the special needs of persons with Autism. Of the educators surveyed, 1 in 3 expressed their interest in utilising an educational module based on simulation-based learning, if available.

### 3.2. Student Surveys

The student surveys assessed student demographics, their experiences with persons with Autism (Table 2), knowledge on Autism (Table 3) and their self-rated efficacy in managing persons with Autism (Table 4). Of the 135 Bachelor of Oral Health Therapy (BOHT) students invited to participate from University of Newcastle, 38 responses were received (28.1% response rate). Of these, 26 complete responses were obtained with 12 participants choosing not to respond to all sections.

Student demographics and their experience of Autism are presented in Table 2. A total of 22 (57.9%) first-year and 16 (42.1%) third-year BOHT students responded to the survey. Most participants were female (84.2%) with an age range between 18 and 41 years. Students had a range of ethnic backgrounds with 44.7% Australian, 15.8% Asian, 7.9% European, 7.9% Middle Eastern, and 23.7% choosing not to disclose their ethnicity. The majority (65.8%) of the respondents had no experiences treating persons with Autism whereas 26.3% of the respondents reported that they did. This may be attributed to the fact that the majority of the students were in their first year (57.8%) and these students are yet to commence clinical sessions involving patients.

The survey results indicated that 84% of the respondents had a good knowledge of Autism as they answered questions related to various aspects of Autism accurately (Table 3). However, only 36.1% of the respondents were aware that persons with Autism are not more prone to interpersonal violence than non-Autistic people and only 50% correctly answered that females are more difficult to diagnose with Autism than males.

In relation to self-rated efficacy, responses received on the 7-point Likert scale were grouped into three levels of confidence (Table 4): low confidence (1–3); moderate confidence (4–6); and high confidence (7). Notably, 25% reported lower confidence levels in recognising the signs and symptoms of children with Autism and 38.5% in adults with Autism. Only one respondent reported a high confidence level in recognising the signs and symptoms of Autism in both children and adults. None of the respondents reported high confidence in treating children or adults with Autism. Additionally, almost half of the respondents (46.2%) reported a moderate level of confidence in awareness around sources of further information and guidance for treatment for persons with Autism along with awareness around the relevant care pathways (42.3%). The majority of the respondents reported a moderate level of confidence in awareness that adjustments could be made to facilitate treatment for persons with Autism (72.0%) and making adjustments in their own practice (68.0%).

### 3.3. Responses to Polar and Open-Ended Questions

The response data comprised 14 “yes” and one “no” responses to Question 1: “*Do you plan to treat persons with ASD as an Oral Health Therapy graduate?*”. Additionally, there were 14 “yes” responses to Question 2: “*Are you interested in attending CPD courses as a graduate to further develop your confidence and competence treating persons with ASD*?” were recorded.

The student responses to the open-ended questions were analysed following Braun and Clarke’s reflexive thematic analysis [21,22]. Patterns were identified across responses within a framework of interpretivism and constructivism. Themes were reviewed for coherence and defined with supporting quotes from students. Six themes emerged for Question 1 and four for Question 2.

Most respondents to the question related to treating persons with Autism expressed a strong ethical commitment to equal care and non-discrimination, emphasising fairness and the principle that individuals with Autism deserve equal treatment. Professional responsibility for an OHT framed ASD care as integral to their scope of practice. Two respondents highlighted ASD as part of the patient population, acknowledging the inevitability of encountering patients with ASD and the growing need for special care services. Adaptation strategies featured prominently in accommodating unique needs and sensory-friendly care with students describing plans to create sensory-friendly environments and the use of gentle communication. Reservations and uncertainty including challenges reflected perceived difficulties in managing cooperation, while personal connection and experience appeared in comments referencing familiarity with individuals with ASD.

Interest in continuing professional development was high, with skill development/knowledge building emerging as the dominant theme. Respondents sought to enhance clinical skills and theoretical understanding to provide effective care. Confidence building was frequently linked to CPD participation, while commitment to inclusive practice within their scope underscored the desire to treat all patients appropriately within professional boundaries. Two respondents expressed interest in diversity and inclusion, extending beyond ASD to broader patient populations. Practical barriers were noted with practical constraints (time, etc.), indicating challenges in balancing CPD with other commitments.

Across both questions, responses reflected a positive and inclusive orientation toward ASD care, with emphasis on ethical responsibility and professional growth. While most students demonstrated readiness to adapt their practice, a minority expressed reservations, suggesting areas for targeted support in future training.

## 4. Discussion

This cross-sectional pilot study assessed the extent of ASD-related content in Australia and New Zealand (ANZ) dental and oral health curricula and explored Oral Health Therapy students’ knowledge and self-efficacy gap in structured teaching on sensory adaptations and communication strategies, despite educators expressing interest in simulation-based learning. Limited use of simulation activities suggests an opportunity to integrate experiential modules that align with best-practice frameworks for special needs dentistry.

Education related to the delivery of oral health care for priority groups has been identified as a requirement by the accrediting body for dental education in Australian Universities, the Australian Dental Council [23]. Furthermore, Australian Dental Council (ADC) professional competencies of the newly qualified dental practitioner (2023) highlights the expectation that dental practitioners must be able to provide oral health care for groups or populations at increased risk of harm or poor oral health and deliver person-centred care [23] for all patients, including persons with Autism. Additionally, a shift within the broader health system to support individuals in making decisions about their own health care is evident [23]. A recent Australian study reviewed the curricula of oral health programmes regarding the oral health care of persons with a disability and identified the need to standardise the clinical training of undergraduates as a critical component in improving oral health care for this population [24]. Our findings demonstrated the willingness of students at the University of Newcastle to learn strategies to adapt their delivery of oral health care to support individuals in making decisions about their own oral health.

The need to educate dental practitioners on the sensory needs and adaptations for persons with Autism has been recognised in the literature as Autism is a common condition [13,25]. Additionally, it is important that health practitioners include details in the patient’s history regarding neurodiversity and sensory sensibilities to be considered when delivering oral health care in a sensory-adapted dental environment [25]. Their study also reported that the involvement of family members prior to and within dental visits facilitated positive outcomes including clinical experiences and aided the development of critical strategies in reducing dental anxiety. Specifically, completion of a pre-treatment questionnaire [25] can be used as a strategy for student clinicians to adapt to the individual patient needs.

Mixed opinions were received in the educator surveys regarding the methodology for teaching special care dentistry in undergraduate education. Some employed role play and face-to-face education while others used visuals and tutorial discussion to share personal experiences around Autism management. Limited utilisation of simulation-based learning in universities in Australia and New Zealand on adapting to the sensory needs of persons with Autism was noted along with acknowledgement that simulation-based education would be beneficial. Furthermore, the educators indicated their interest in utilising an educational module on Autism management in the dental setting. Although the response rate in these surveys was low, the findings align with previous studies that show that the undergraduate education for medical and dental students in the delivery of preventive health care for persons with Autism needs improvement [26,27].

Traditionally, simulation-based learning experiences include scenarios for simulation and written activities, use of mannequins, hi-fidelity patient simulators, role play and immersive simulation using part-task trainers [17,28]. Many teaching facilities lack resourcing for hi-fidelity simulation equipment and often utilise scenario-based solutions using existing facilities. Hybrid simulation facilitates a combination of scenarios including human interactions, reactions and non-verbal cues from body language to develop both procedural and communication skills [28]. Using a hybrid simulation activity provides a standardised patient experience for students and can utilise actors or peer role modelling to simulate the realism of the training by providing the feelings and emotions of the experience [28,29]. The transition from simulation to the clinical environment can be facilitated by incorporating hybrid simulation-based education with observational placements and finally by introducing the student(s) to the real-life patient experience of treating patients [28]. Such a model would effectively increase the proportion of dental professionals able to provide preventive focused special needs dentistry. This can also reduce the barriers identified by parents, carers and Autistic patients for accessing dental care [10].

Quantitative analysis of student responses provided further insight with over 63% of respondents demonstrating good knowledge of Autism, indicating a reasonable theoretical foundation. However, misconceptions did persist, as just over one in three students (36%) correctly identified that persons with Autism are not more prone to interpersonal violence than neurotypical individuals. This suggests that while general awareness exists, nuanced understanding of Autism-related facts requires reinforcement through targeted education. Self-efficacy results from our study showed that 71% of students reported moderate confidence in recognising signs and symptoms of Autism in children, compared to 58% in adults. High confidence was rare, with only one respondent reporting strong ability to identify Autism characteristics, and none reporting high confidence in treatment delivery, highlighting a gap between theoretical knowledge and perceived clinical competence. Confidence in practical adjustments was somewhat better, with 72% moderately confident in incorporating optimal adaptations and 68% in implementing these changes. These findings underscore that knowledge alone does not translate into clinical confidence, reinforcing the need for experiential learning opportunities such as simulation-based modules and patient interactions.

The thematic analysis provides important insights into the attitudes and professional development needs of students on care for persons with Autism. The findings demonstrate a strong ethical commitment to inclusive care and recognition of ASD as part of the patient population. This is highly relevant to oral health education, as it supports the integration of person-centred care principles and highlights the need for targeted training to address neurodiverse patient needs. The expressed interest in continuing professional development (CPD) underscores the importance of embedding Autism-related competencies within curricula and offering accessible post-graduate learning opportunities.

The open-ended responses from students indicated that they were keen to learn to adapt their approach to the provision of oral health care to the needs of persons with Autism and special needs, whereas one student commented that it would be “*too difficult to engage with them if they are unwilling to cooperate*”. Knowledge of Autism and understanding of the sensory and medical needs of persons with Autism are needed to deliver person-centred oral health care. It is important for dental students to be aware of ways to adapt their clinical practice to the sensory and medical needs of persons with Autism to address the barrier to oral health for this population of patients [10]. However, other students commented that it would be “*discriminatory not to. If they need help, I’ll do it no matter who it is*”.

Our findings suggest that oral health programmes should prioritise experiential learning and CPD pathways focused on managing persons with Autism. It has been shown in the literature that dental curricula need to be developed to include structured, experiential training to ensure that graduates are equipped to deliver person-centred care for all patient populations including persons with disabilities [30]. Future research could explore the effectiveness of such interventions and examine whether stated intentions translate into clinical practice. Longitudinal studies and multi-site samples would strengthen evidence and inform policy for inclusive oral health care. A strength of this study lies in its mixed-methods approach, combining quantitative measures with qualitative insights that capture attitudes and motivations beyond numerical trends. The use of Braun and Clarke’s reflexive thematic analysis adds rigour and transparency. However, limitations include a small sample size from a single institution (students), potential social desirability bias, and brief responses that constrained interpretive depth.

There are some notable limitations in this cross-sectional study, including the low response rates, particularly within the survey involving educators. Educator recruitment via professional bodies (COHA/ACODS) and voluntary student participation may introduce selection and non-response bias suggesting a cautious interpretation of results. Additionally, the student surveys were conducted using a convenience sampling approach with a single university programme (University of Newcastle) which suggests potential response bias due to limited external validity.

Furthermore, there were multiple questions within both the surveys that the respondents chose to skip, resulting in limited power in drawing conclusions based on the reported findings. Future studies could improve response rates through a combination of methodological, procedural and engagement-focused strategies. Targeted and personalised recruitment approaches, such as direct invitations from known institutional leaders in education or course coordinators, may increase perceived relevance and legitimacy. Offering appropriate incentives, such as professional development recognition, certificates, or small prizes, has been shown to increase motivation [31]. These strategies may enhance participation, reduce non-responsive bias, and strengthen the validity and generalizability of future findings.

## 5. Conclusions

Within the constraints of low response rates and a single institution student sample, these preliminary findings suggest opportunities to strengthen Autism-related teaching, particularly sensory adaptations, communication strategies, and experiential learning. This cross-sectional pilot study indicates that Autism-specific training in Australia and New Zealand dental and oral health programmes may be limited and that students, while demonstrating foundational knowledge, report modest self-efficacy for clinical management. These findings should be viewed as preliminary and hypothesis generating observations rather than definitive assessment of curricula across Australia and New Zealand. Future multi-site studies with higher response rates, validated instruments and objective curriculum audits are warranted which should include evaluation of experiential education such as simulation.

## Figures and Tables

**Table 1:** Response(s) from educators (members of COHA and ACODS).

**Question**	Yes (%)	No (%)	No Reply/ Don’t Know (%)	Total (%)
Does your Dentistry or Oral Health curriculum include education for students in the treatment and management of persons with Autism?	7 (46.7%)	5 (33.3%)	3 (20.0%)	15 (100%)
Are you be interested in using an educational module to teach dental practitioners about Autism in the dental setting?	5 (33.3%)	0 (0.0%)	10 (66.7%)	15 (100%)
How many hours (approx.) during their studies would this include? Responses: 2 h, 2.5 h	2 (13.3%)	0 (0.0%)	13 (86.7%)	15 (100%)
Face to Face Delivery of Education Responses: 1 h lecture in BOH2 paediatrics, 1.5 h tutorial on the neurodivergent patient in BOH3 integrated theory	5 (33.3%)	0 (0.0%)	10 (66.7%)	15 (100%)
Online Synchronous (Live) Delivery of Education	2 (13.3%)	3 (20.0%)	10 (66.7%)	15 (100%)
Asynchronous (Recorded) Delivery of Education	2 (13.3%)	3 (20.0%)	10 (66.7%)	15 (100%)
Other Delivery of Education Responses: Role play	1 (6.7%)	0 (0.0%)	14 (93.3%)	15 (100%)
Are you familiar with a Sensory Adapted Dental Environment (SADE)?	3 (20.0%)	2 (13.3%)	10 (66.7%)	15 (100%)
Do you teach the topics listed below?				
SADE for persons with Autism	2 (13.3%)	1 (6.7%)	12 (80.0%)	15 (100%)
Communication for persons with Autism	4 (26.7%)	0 (0.0%)	11 (73.3%)	15 (100%)
Oral hygiene instruction for persons with Autism	2 (13.3%)	1 (6.7%)	12 (80.0%)	15 (100%)
Pretreatment questionnaires for persons with Autism	2 (13.3%)	1 (6.7%)	12 (80.0%)	15 (100%)
Developing social stories for persons with Autism	1 (6.7%)	2 (13.3%)	12 (80.0%)	15 (100%)
Role Play for persons with Autism	2 (13.3%)	1 (6.7%)	12 (80.0%)	15 (100%)
Other activity for persons with Autism Responses: Tutorial discussions, sharing personal experiences, visuals	2 (13.3%)	0 (0.0%)	13 (86.7%)	15 (100%)
Does your department deliver Oral Health Simulation Education in the treatment and management of Autistic Individuals?	1 (6.6%)	4 (26.6%)	10 (66.6%)	15 (100%)
Would you consider Oral Health Simulation Education in the treatment and management of Autistic Individuals beneficial?	2 (13.3%)	2 (13.3%)	11 (73.3%)	15 (100%)

**Table 2:** Response rate and demographics of the students.

Question	Details	Responses (Percentage)
Student cohort	First-year BOHT	22 (57.9%)
	Third-year BOHT	16 (42.1%)
Demographics	Female	32 (84.2%)
	Male	2 (5.3%)
	Did not answer	4 (10.5%)
Country of origin	Australian	17 (44.7%)
	Asian	6 (15.8%)
	Europeans	3 (7.9%)
	Middle East	3 (7.9%)
	Unknown/Not stated	9 (23.7%)
How many patients with Autism have you treated?	≥10 persons	3 (7.9%)
	5–9 persons	4 (10.5%)
	1–4 persons	6 (15.8%)
	Did not answer	25 (65.8%)
Personal experience with Autism	Yes	10 (26.3%)
	No	19 (50.0%)
	Did not answer	9 (23.7%)

**Table 3:** Responses from the students on knowledge questions.

True/False Questions	True (%)	False (%)	No Reply (%)	Total (%)
People with Autism can be interested in social interaction	25 * (65.8%)	4 (10.5%)	9 (23.7%)	38 (100%)
Independent living is not possible for Autistic people	1 (2.6%)	28 * (73.7%)	9 (23.7%)	38 (100%)
People with Autism feel no empathy or affection	1 (2.6%)	28 * (73.7%)	9 (23.7%)	38 (100%)
A lack of eye contact is necessary for a person to be considered Autistic	2 (5.3%)	27 * (71.0%)	9 (23.7%)	38 (100%)
Autism cannot be diagnosed in adulthood	1 (2.6%)	28 * (73.7%)	9 (23.7%)	38 (100%)
Most people with Autism also have intellectual disabilities	8 (21.0%)	21 * (55.3%)	9 (23.7%)	38 (100%)
Females are more difficult to diagnose with Autism than males	19 * (50.0%)	10 (26.3%)	9 (23.7%)	38 (100%)
People with Autism always display challenging behaviours	8 (21.0%)	21 * (55.3%)	9 (23.7%)	38 (100%)
Autistic people have difficulty with non-literal language and non-verbal communication (e.g., body language and gesturing)	24 * (63.2%)	4 (10.5%)	10 (26.3%)	38 (100%)
Additional mental health conditions (e.g., anxiety, depression) are more prevalent in individuals diagnosed with Autism than in the general population	24 * (63.2%)	4 (10.5%)	10 (26.3%)	38 (100%)
People with Autism can show unusual reactions to sensory experiences (e.g., lights, touch, sounds, etc.)	27 * (71.1%)	1 (2.6%)	10 (26.3%)	38 (100%)
Autism is a very rare condition, affecting only 0.05% of the Australian population	1 (2.6%)	27 * (71.0%)	10 (26.3%)	38 (100%)
Autistic people are more prone to interpersonal violence than non-Autistic people	15 (39.5%)	13 * (34.2%)	10 (26.3%)	38 (100%)
Change in routine and uncertainty are often upsetting for Autistic people	27 * (71.1%)	1 (2.6%)	10 (26.3%)	38 (100%)
More than half of people diagnosed with Autism do not talk	7 (18.4%)	21 * (55.3%)	10 (26.3%)	38 (100%)

* indicates correct answer.

**Table 4:** Self-efficacy of the confidence levels in students.

Likert Scale	1–3 (Low)	4–6 (Moderate)	7 (High)	Total Responded
Recognising the signs and symptoms of Autism in children	6 (25.0%)	17 (70.8%)	1 (4.2%)	24
Recognising the signs and symptoms of Autism in adults	10 (38.5%)	15 (57.7%)	1 (3.8%)	26
Treating Autistic children	9 (37.5%)	15 (62.5%)	0 (0%)	24
Treating Autistic adults	6 (25.0%)	18 (75.0%)	0 (0%)	24
Knowing where to find further information and guidance for treating Autistic people	14 (53.8%)	12 (46.2%)	0 (0%)	26
Knowing what adjustments could be made to facilitate treatment for Autistic people	7 (28.0%)	18 (72.0%)	0 (0%)	25
Making adjustments in practice to facilitate treatment for Autistic people	8 (32.0%)	17 (68.0%)	0 (0%)	25
Knowing the relevant local care pathways/services for Autistic people	15 (57.7%)	11 (42.3%)	0 (0%)	26

The scale is from 1 to 7 (1 = not at all confident; 4 = somewhat confident; and 7 = extremely confident).

**Table 5:** Quotes received from students (Question 1) “*Do you plan to treat persons with ASD as an Oral Health Therapy graduate?*”.

Response	Quotes/Subthemes (As Superscript)
Yes	I plan to treat persons with ASD as an Oral Health Therapy graduate. I genuinely believe in creating a warm and supportive atmosphere that acknowledges their unique needs. By using gentle communication and personalized approaches, I hope to build trust and make them feel at ease during their visits. My goal is to provide compassionate care that not only addresses their oral health but also makes them feel understood and valued. ^(3)^
Yes	Definitely it would involve understanding and accommodating the unique needs of individuals with ASD. ^(3)^ This includes creating a sensory-friendly environment, communicating clearly, and being patient and flexible during appointments. My role would also involve educating patients and caregivers about oral hygiene practices and making necessary adjustments to ensure comfort and successful treatment outcomes.
Yes	I think it’s important. A person with ASD needs dental care just as someone that does not have ASD. ^(1)^
Yes	They represent a portion of the public so I will undoubtedly treat them. ^(2)^
Yes	There is a large demand for dentistry with special needs. OHT as a preventive provider will play a significant role in treating people with ASD. ^(2)^
Yes	Not as in I plan to, but not opposed. ^(5)^
Yes	It is extremely important for persons with ASD to maintain adequate oral hygiene and receive the treatment that they need. ^(4)^
Yes	If I have chance, I will try my best to helps them. ^(4)^
Yes	I want to provide the care I am able to for all patients equally. ^(1)^
Yes	I know someone with autism, and they are not that different than a regular person, he sometimes doesn’t pick up on social cues or prefers to be by himself but other than that no difference. ^(6)^
Yes	I am interested in working with children from different diversities. ^(1)^
Yes	Discriminatory not to. If they need help, I’ll do it no matter who it is. ^(1)^
Unstated	Because individuals with autism deserve to be treated equally. ^(1)^
Yes	As a health care professional, I am open to treating any patient who needs care, including patients with ASD. ^(1,4)^
No	Too difficult to engage them if they’re unwilling to cooperate. ^(5)^

^1^ Equal care/non-discrimination; ^2^ ASD as part of patient population/demand; ^3^ accommodating unique needs and sensory-friendly care; ^4^ professional responsibility/OHT role; ^5^ reservations/uncertainty/challenges; ^6^ personal connection/experience.

**Table 6:** Quotes received from students (Question 2)—“*Are you interested in attending CPD courses as a graduate to further develop your confidence and competence treating persons with ASD?*”.

Response	Quotes/Subthemes (As Superscript)
Yes	I’m always eager to increase my knowledge and understanding. Attending CPD courses is a great opportunity to further develop my confidence and competence in treating persons with ASD, and I look forward to learning more about how to provide the best care for them. ^(1)^
No	No comment
Yes	I would like to feel more confident in treating people with ASD. ^(1,2)^
Yes	No comment
Yes	It’s critical to empower myself with theoretical knowledge in full scope of treating different types of people. ^(1)^
Yes	I think that learning about different disabilities is a necessity for people finishing a degree to ensure all people can be treated effectively and appropriately. ^(1,2)^
Yes	To develop confidence and know how and when to treat them
Yes	Treat different patients is also helpful for us to develop our communication skills and can help them get better treatment. ^(1)^
Yes	To develop my knowledge and clinical skills further which enables a more comfortable environment. ^(1)^
Yes	To develop my knowledge and clinical skills further which enables a more comfortable environment. ^(1)^
Yes	I have a strong interest in working with children/adult from all different background and diversity. ^(1,3)^
Yes	I’d like to but struggle with finding time for things. ^(4)^
Yes	As an OHT, I would like to be able to treat individuals with autism and be confident in making them feel comfortable in the practice. ^(3)^
Yes	To improve my understanding and become a better healthcare professional. ^(1)^
Yes	Will be good to know how to treat them if there’s patients with ASD. At least I have an idea how to treat them and try my best to do it if they come to the clinic. ^(1)^

^1^ Skill development/knowledge building; ^2^ confidence building; ^3^ interest in diversity and inclusion; ^4^ practical constraints (time, etc.).

## Data Availability

The raw data supporting the conclusions of this article will be made available by the authors on request.

## Supplementary Materials

The following supporting information can be downloaded at: https://www.mdpi.com/article/10.3390/dj14040238/s1, STROBE Statement Checklist Cross-Sectional Studies (Completed).
