# Supplementary material for: Evaluating Undergraduate Dental Curricula on Oral Health Care for Autistic Persons in Australia and New Zealand—A Cross-Sectional Study
Source: Dent J (Basel). 2026 Apr 15;14(4):238. doi: 10.3390/dj14040238 (PMC13114377; doi:10.3390/dj14040238)
Supplement: Supplementary file 1 [file dentistry-14-00238-s001.zip › dentistry-4163328-supplementary.pdf]

STROBE Statement Checklist Cross-Sectional Studies (Completed)

Manuscript: Evaluating Undergraduate Dental Curricula on Oral Health Care for Autistic Persons in Australia and New Zealand – A Cross-Sectional Study

	Item No	Recommendation
**Title and abstract**	1	Study design with a commonly used term in the title and the abstract
		Abstract includes an informative and balanced summary with limitations
**Introduction**		
Background/rationale	2	Literature background content and rationale for the investigation is reported
Objectives	3	Specific objectives stated in Introduction
**Methods**		
Study design	4	Described in methods
Setting	5	Described the setting, locations, and relevant dates, including periods of recruitment, and data collection
Participants	6	Eligibility and recruitment sources described (Methods – Educator and Student Surveys)
Variables	7	Outcomes and measures described (methods – Instruments)
Data sources/ measurement	8	Surveys via RedCap®; descriptive analysis: reflexive thematic analysis (Methods)
Bias	9	Selection and non-selection bias discussed (Methods/Discussion)
Study size	10	All available responses, pilot study; no formal power calculation (Methods)
Quantitative variables	11	Descriptive summaries: Likert grouping described (Method/Results)
Statistical methods	12	Descriptive statistics only; qualitative thematic analysis (methods)
**Results**		
Participants	13	Flow and denominators reported (Results/tables)
Descriptive data	14	Demographics and experience (Results/Table 2)
Outcome data	15	Knowledge and Self-efficacy (Results/tables 3-4)
Main results	16	Cautious interpretation without causal claims (Results/Discussion)
Other analyses	17	Qualitative Themes reported (Tables 5-6; Figures 1-2)
**Discussion**		
Key results	18	Summarised key results in Discussion.
Limitations	19	Expanded and emphasised (Discussion/Conclusion)
Interpretation	20	Placed in context; moderate claims (Discussion)
Generalisability	21	Acknowledges small sample size and limited external validity of the study
**Other information**		
Funding	22	Not Applicable
